# A novel 7 RNA-based signature for prediction of prognosis and therapeutic responses of wild-type BRAF cutaneous melanoma

**DOI:** 10.1186/s12575-022-00170-2

**Published:** 2022-06-24

**Authors:** Ruizheng Sun, Yaozhong Liu, Cheng Lei, Zhenwei Tang, Lixia Lu

**Affiliations:** 1 Department of Dermatology, Xiangya Hospital, Central South University, 87# Xiangya Road, Changsha, 41008 Hunan China; 2 Clinical Medicine Eight-Year Program, Central South University, Changsha, China; 3 Department of General Surgery, Xiangya Hospital, Central South University, Changsha, Hunan China; 4 Department of Cardiology, the Second Xiangya Hospital, Central South University, Changsha, Hunan China; 5 Hunan Key Laboratory of Skin Cancer and Psoriasis, Xiangya Hospital, Central South University, Changsha, Hunan China; 6 Hunan Engineering Research Center of Skin Health and Disease, Changsha, Hunan China; 7 National Clinical Research Center for Geriatric Disorders, Xiangya Hospital, Changsha, Hunan China

**Keywords:** BRAF, Melanoma, Signature, TCGA, mTOR, Prognosis

## Abstract

**Background:**

The prognosis of wild-type BRAF cutaneous melanoma (WT Bf-CM) patients remains poor due to the lack of therapeutic options. However, few studies have investigated the factors contributing to the prognosis of WT Bf-CM patients.

**Methods:**

In this paper, we proposed and validated a novel 7-RNA based signature to predict the prognosis of WT Bf-CM by analyzing the information from TCGA database.

**Results:**

Dependence of this signature to other clinical factors were verified and a nomogram was also drawn to promote its application in clinical practice. Functional analysis suggested that the predictive function of this signature might attribute to the prediction of the up-regulation of RNA splicing, transcription, and cellular proliferation in the high-risk group, which have been demonstrated to be linked to malignancy of cancer. Moreover, functional analysis and therapy response analysis supported that the prognosis is highly related to PI3K/Akt/mTOR pathway among WT Bf-CM patients.

**Conclusion:**

Collectively, this study will provide a preliminary bioinformatics evidence for the molecular mechanism and potential drug targets that could improving WT Bf-CM prognosis.

**Supplementary Information:**

The online version contains supplementary material available at 10.1186/s12575-022-00170-2.

## Introduction

Characterized by rapid progression and metastasis, cutaneous melanoma (CM) is considered as the most aggressive skin cancer, accounting for about 55,500 death worldwide annually [1]. During the past decades, some breakthroughs have been made and significantly improve the survival of metastatic CM patients. For example, the clinical application of BRAF inhibitors and MEK inhibitors, which can extend the progression-free survival (PFS) of BRAF V600-mutate CM patients to 11–14.9 months [1]. However, CM patients carrying BRAF V600 mutations only makes up 45–50% of total melanoma patients [2]. For wild-type BRAF CM (WT Bf-CM) patients, that accounts for about 30% of total CM patients, the response rate to MEK inhibitor is merely 10% [3–5]. Lack of therapeutic options resulted in the poor prognosis of WT Bf-CM. With a chemotherapy of dacarbazine, the median PFS of WT Bf-CM patients can be low as 1.5 months [6].

Recently, with the help of next-generation sequencing (NGS) technology, the high heterogeneity of CM has been demonstrated, and it turns out that some features of this heterogeneity such as tumor mutational burden, are associate with the prognosis of CM [7]. Moreover, the high heterogeneity of CM indicates the existence of various biomarkers for prognosis. Although studies have revealed different biomarkers or signature for survival prediction, few of them have focused on the patients with wild-type BRAF CM [8–10]. Little is known about which factors can contribute to the poor prognosis of WT Bf-CM patients, apart from less therapy options, failing the need of precise management of WT Bf-CM patients.

To address this issue, this paper analyzed the whole transcription profiles of tumor tissues from 211 patients in the TCGA database, and proposed and validated a novel 7-RNA based signature to predict the prognosis of with wild-type BRAF CM. Moreover, functional analysis and therapy response analysis were also performed, providing a preliminary bioinformatics evidence for the molecular mechanism and potential drug targets that could improving WT Bf-CM prognosis.

## Materials and methods

### Data extraction

The transcriptional profiles, mutation data and corresponding clinical information of 467 CM patients were downloaded from TCGA-SKCM project with an R package “TCGAbiolinks” [11]. After excluding patients with BRAF somatic mutations and incomplete survival information, 211 WT Bf-CM patients’ tumor samples were equally allocated into training group (*n* = 106) and testing group (*n* = 105). NRAS and NF-1 somatic mutational statuses of WT Bf-CM patients were extracted from mutation data. We also downloaded DNA methylation status of specific sites of the WT Bf-CM patients using R package “cgdsr”. RNA-seq data were alignment to the human genome (Gencode.v22 annotation) and the expression of RNAs were measured by Reads Per Kilobase per Million mapped reads (RPKM). RNA expression profiles were identified based on the two following criteria: 1) transcripts were expressed in all WT Bf-CM samples; 2) average of RPKM > 0.1. Eventually, a total of 14,202 RNAs in the profiles were enrolled.

### Statistical analysis

In the training set, correlations between the expression of each RNA and overall survival (OS) of patients were calculated by univariate Cox regression and 251 RNAs with their *P*-values < 0.01 were considered significant to patients’ OS. Random forests were grown based on survival-related RNAs and variable hunting was implemented repeatedly using R package “randomForestSRC”, which output top ranked variables by frequency of occurrence. 7 OS-relevant characteristics were selected as the best and fitted into stepped multivariate Cox regression analysis. Risk scores were weighted by the expression and coefficients of 7 RNAs. The median of risk scores in the training set served as a cut-off, respectively dividing WT Bf-CM patients into high risk groups and low risk groups both in the training set and the testing set. Kaplan–Meier (KM) analysis and visualization were performed to evaluate the survival differences between high risk groups and low risk groups by the “survival” R package. For WT Bf-CM patients with at least 5-year follow ups, receiver operating characteristic (ROC) curves were used to show the sensitivities and specialties of signatures’ abilities to predict the 5-years OS using categorical variables. The area under ROC (AUC) scores and 95% confidence interval (CI) to estimate the reliabilities of signatures were calculated by R package “pROC” [12]. To demonstrate the overview information of risk scores, patients’ survival and expression of signature genes, data were listed in descending order by scaled risk scores and showed through R packages “ggplot2” and “heatmap” as previously described [13]. The results of univariable and multivariable Cox analysis of 7-RNA signature and clinical variables were visualized by the R package “forestplot”.

### Nomogram and Decision Curve Analysis (DCA)

Three factors including our signature scores, age and tumor stage which were tightly associated with patients’ survival in multivariable Cox analysis were selected, integrated and used to establish a new model predicting 3-year and 5-year survival of WT Bf-CM patients. Nomogram was drawn with the help of R package “rms”. C-index and calibration curve were used to evaluate the predicting ability of nomogram. DCA is an effective method to estimate molecular signatures and prediction models, especially considering decision preference and clinical demands [14]. Visualization of DCA were achieved in the R statistics environment with the guide of “stdca” (https://github.com/matt-black/dcapy/blob/master/test/resource/stdca.R).

### Functional analysis

To explore the function implications of the 7-RNA signature, the differently expression analysis between high risk groups and low risk groups were carried out using R package “edgeR” [15]. Differently expressed genes (DEG) were defined as absolute value of log_2_FoldChange > 0.8 and adjusted *p* values < 0.001. The potential interactions of 527 protein-coding DEGs were estimated in String and laid out using Cytoscape [16, 17]. The application in Cytoscape Mcode was used to find the core gene module using criteria of Mcode scores [18]. The top biological process (BP) enrichment results of all DEGs and expression of involved genes were demonstrated and clustered by BP terms using R packages “GOplot” [19]. Gene set enrichment analysis (GSEA) on interested enrichment sets in MSigDB was based on R package “clusterProfiler” [20].

### Immune infiltration and drug prediction

To investigate the immune microenvironment of WT Bf-CM tumor samples, 24 sorts of immune cells of each sample were quantified using single sample GSEA (ssGSEA) based on marker genes in previous study and immune infiltration was evaluated by the results of Hierarchical cluster [21, 22]. Combination of immune infiltration results and clinical features was demodnstrated using R package “ComplexHeatmap” [23]. As previously described, we used an R package “pRRophetic” to predict the inhibitory concentration 50 (IC50) of various anti-tumor drugs based on expression profiles [24].

## Results

### Clinical characteristics of the study populations

A total of 211 WT Bf-CM patients’ samples was downloaded from TCGA. As is shown in Table 1, 64.0% of the samples were collected from male and 36% were from female. The median age of this cohort is 62, and the range of age is 25 ~ 87. 193 samples were matched with pathological stages according to the American Joint Committee on Cancer (AJCC) Cancer staging manual, the percentages of stage 0, I, II, III, IV were 1.9%, 16.6%, 29.9%, 39.3% and 3.8%, respectively. 25.5% of these samples featured with a Breslow thickness < 2 mm, 28.5% within the range of 2 mm to 5 mm, 25.1% > 5 mm. More information of the selected clinical characteristics of are listed in Table 1.

**Table 1:** Clinico-pathological characteristics of WT Bf-CM patients

Groups	Total (*N* = 211)	Training Group (*N* = 106)	Test Group (*N* = 105)
**Gender**			
Male	135(64.0%)	73(68.9%)	62(59.0%)
Female	76(36.0%)	33(31.1%)	43(41.0%
**Age At Diagnosis**			
Median	62	63.5	61
Range	25 ~ 87	30 ~ 87	25 ~ 83
≤ 58	90(42.7%)	42(39.6%)	48(45.7%)
> 58	121(57.3%)	64(60.3%)	57(54.3%)
**Tumor Tissue Site**			
Primary Tumor	38(18.0%)	20(18.9%)	18(17.10%)
Regional Cutaneous Or Subcutaneous Tissue	38(18.0%)	21(19.8%)	17(16.20%)
Regional Lymph Node Metastasis	93(44.1%)	46(43.4%)	47(44.80%)
Distant Metastasis	40(19.0%)	18(17.0%)	22(21.00%)
Unknown	2(0.9%)	1(0.9%)	1(1.00%)
**Pathological Stage**			
0	4(1.9%)	1(0.9%)	3(2.90%)
I	35(16.6%)	13(12.3%)	22(21.00%)
II	63(29.9%)	33(31.1%)	30(28.60%)
III	83(39.3%)	43(40.6%)	40(38.10%)
IV	8(3.8%)	4(3.8%)	4(3.80%)
Unknown	18(8.5%)	12(11.3%)	6(5.70%)
**Anatomic Site**			
Head And Neck	16(7.6%)	8(7.5%)	8(7.60%)
Extremities	108(51.2%)	55(51.9%)	53(50.50%)
Trunk	56(26.5%)	28(26.4%)	28(26.70%)
Others/Unknown	31(14.7%)	15(14.2%)	16(15.20%)
**Breslow Thickness** (mm)			
< 2	54(25.5%)	26(24.6%)	28(26.70%)
2 ~ 5	60(28.5%)	36(33.9%)	24(22.80%)
> 5	53(25.1%)	25(23.6%)	28(26.70%)
Unknown	44(20.9%)	19(17.9%)	25(23.80%)
**Radiation Therapy**			
Yes	37(17.5%)	20(18.9%)	17(16.20%)
No	155(73.5%)	75(70.8%)	80(76.20%)
Unknown	19(9.00%)	11(10.40%)	8(7.60%)
**Vital Status**			
Yes	81(38.4%)	36(34.0%)	45(42.90%)
No	130(61.6%)	70(66.0%)	60(57.10%)

### Construction and validation of the expression-based prognostic signature

In order to generate the training set and test set, 211 WT Bf-CM patients were divided equally into two set at random, and their clinical characteristics are shown in Table 1. By analyzing the transcriptome data of training set with univariate Cox regression, 251 RNA were shown significantly to correspond with the 5-year OS of WT Bf-CM patients (*p* < 0.01). Next, multivariate Cox stepwise regression was performed based on the results of random forests and an expression-based prognostic signature was constructed. This prognostic signature is based on the hazard ratio of 7-RNA and can be formulated as: Risk scores = 0.318*expression of CDC73 + 0.282*expression of RP1-69E11.3 + 0.882*expression of RP11-188D8.1 + 0.968*expression of RP11-116P24.2 + 0.486*expression of TRIB2 + 0.203*expression of VPS13D + 0.199*expression of CELF3. More information about the 7 RNA is listed in Table 2.

**Table 2:** 7 RNAs significantly associated with the overall survival

Gene symbol	Gene ID	Gene_type	Coeffcient	HR	*p* value
CDC73	ENSG00000134371	protein coding	0.318	1.374	0.015
RP1-69E11.3	ENSG00000237131	processed pseudogene	0.282	1.326	0.044
RP11-188D8.1	ENSG00000271427	lincRNA	0.882	2.416	0.013
RP11-116P24.2	ENSG00000281535	lincRNA	0.968	2.633	0.040
TRIB2	ENSG00000071575	protein coding	0.486	1.625	0.012
VPS13D	ENSG00000048707	protein coding	0.203	1.225	0.034
CELF3	ENSG00000159409	protein coding	0.199	1.220	0.108

**Fig. 1:**
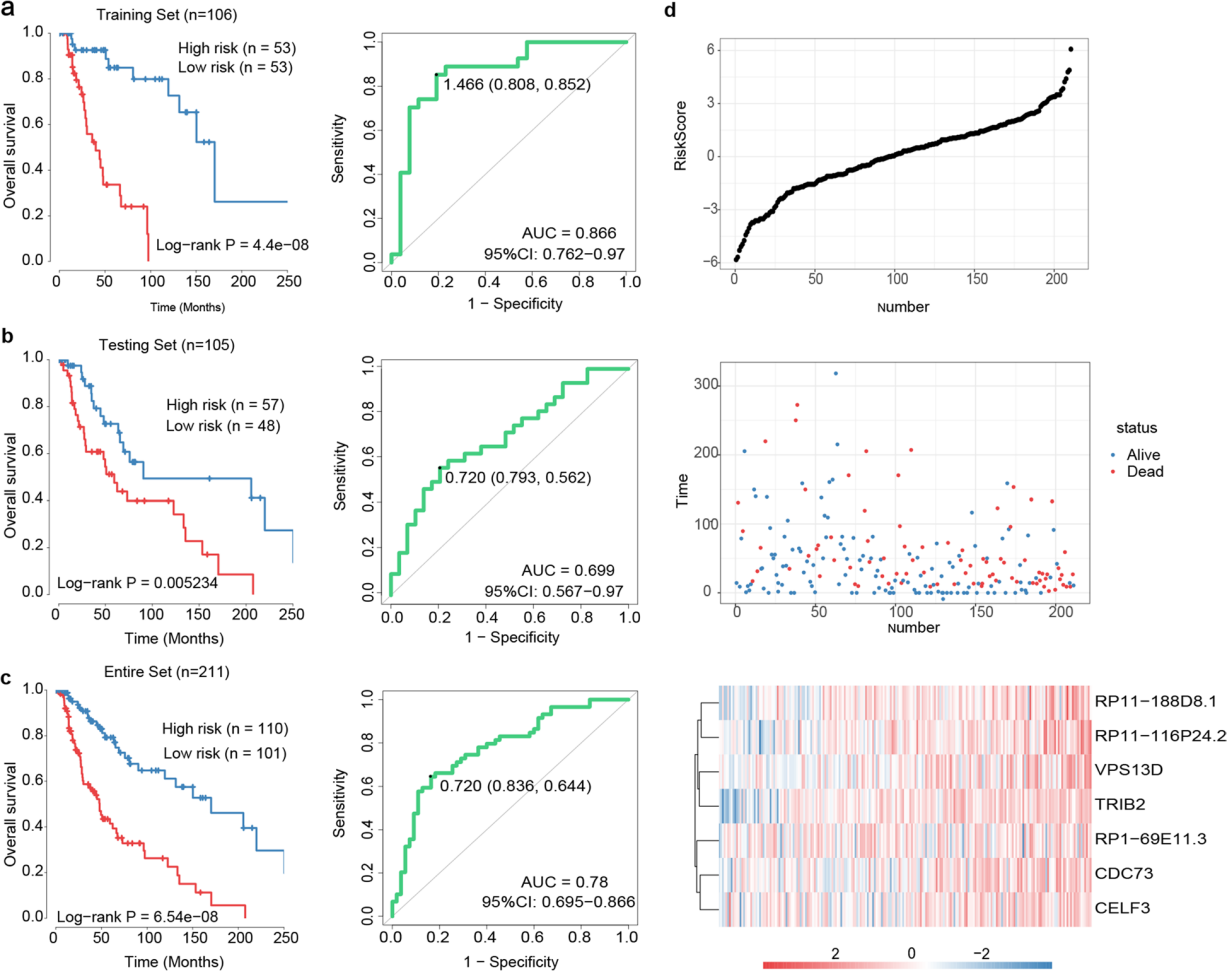
The 7-RNA signature-related risk score and OS prediction of WT Bf-CM patients. KM analysis reveal the OS differences between high and low risk groups and ROC curves assess the predictive performance of the signature-related score in training set (**a**); in testing set (**b**) and in the entire set (**c**). *P* values were calculated by two-sided log-rank tests, total AUC values were estimated and 95% CI were computed with 2000 stratified bootstrap replicates. **d** Risk-related plots illustrate the risk scores, survival status of patients and heatmap of 7 RNAs’ expression

The risk scores of each patient from training set were calculated and the median score was used as cut-off for the stratification of high risk group and low risk group. Kaplan–Meier survival analysis and Log-rank test of this two groups showed a significantly poorer prognostics of high risk group compared to low risk group (39.8 months vs. 170.2 months, *p* < 0.001) (Fig. 1a). For the prognostic value of the proposed signature, ROC curve of training set was drawn and AUC was calculated to be 0.866 (Fig. 1a). To further validate the performance of the proposed signature, patients from test set were stratified similarly based on the median score from training set. Same analysis and tests were also performed with the high- and low-risk groups of test set. Besides the significant correspondence between risk-score and prognosis (59.3 months vs. 90.4 months, *p* = 0.005), the ROC curve (AUC = 0.699) reveals the considerable potential of this signature for predicting the prognosis of WT Bf-CM patients in the test set (Fig. 1b). Similar results were also observed in the entire WT Bf-CM set that high risk group had shorter OS than low risk group (47.9 months vs. 170.2 months, *p* < 0.001) and the AUC was 0.780 (Fig. 1c). Risk-related profiles revealed that more deaths occurred and expression of signature genes increased with the raise of risk scores (Fig. 1d).

### Independence evaluation of the signature in prognostic prediction from clinical and pathological factors

**Fig. 2:**
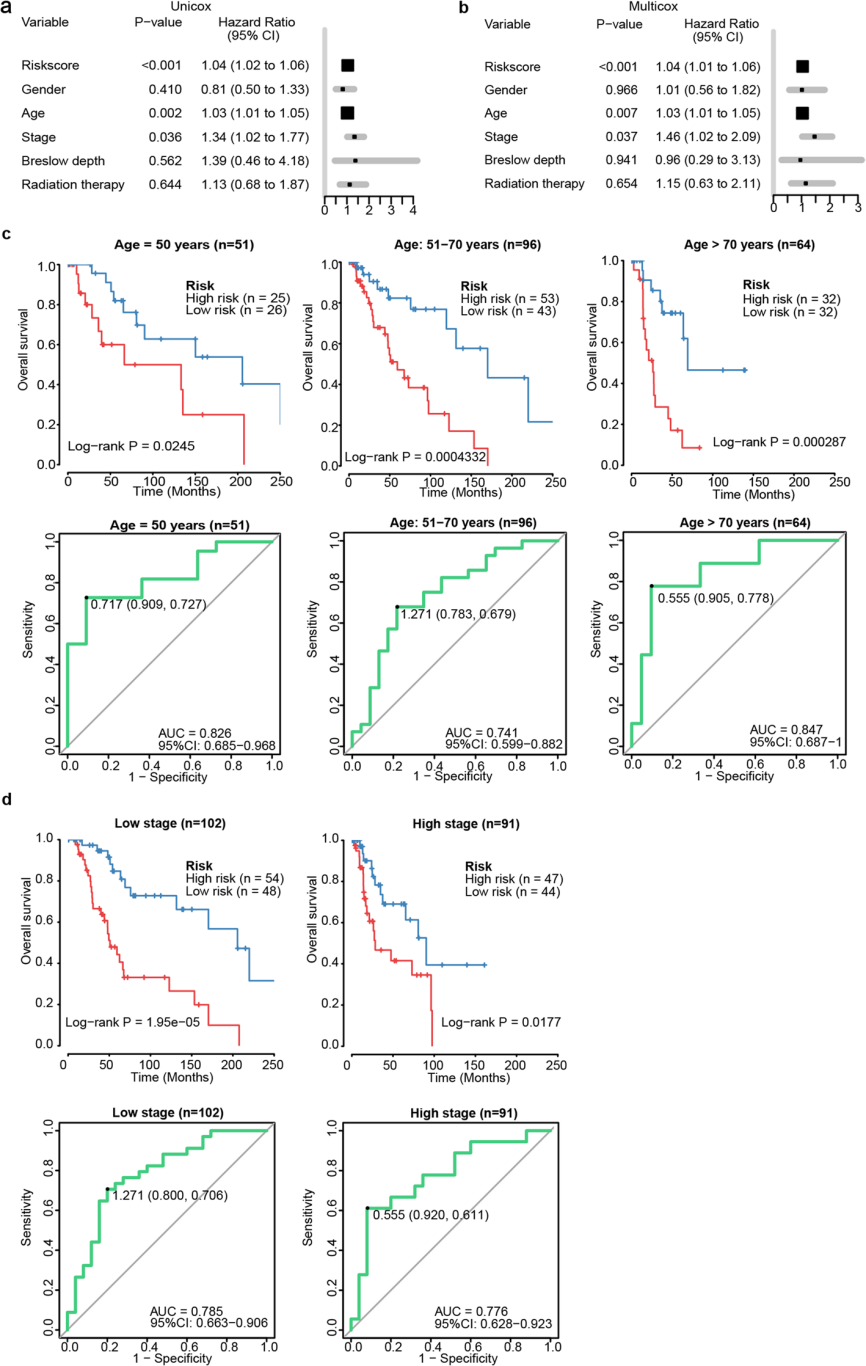
Signature’s independence of clinical factors and stratification analysis. Univariable Cox regression and multivariable Cox regression analysis were performed, *P*-value (significance), Hazard Ratio and 95% CI were respectively shown in (**a**) and (**b**). KM and ROC analysis of regrouping cohorts based on age and stage were correspondingly demonstrated in (**c**) and (**d**)

Traditional clinical and pathological factors have been well-clarified to be associated with the prognosis of melanoma patients, including age, gender, pathological stage, Breslow depths and radiation therapy [1, 25]. Using univariable and multivariable analysis, age and stage were shown to be significantly associated with the prognosis of 211 WT Bf-CM patients in addition to the proposed signature (Fig. 2a). Therefore, age and stage were chosen to be compared with the proposed signature for its independence. As is shown in Fig. 2b, when applied in each subgroup of entire set divided according to the risk stratification of each factor, the proposed signature revealed considerable performances in prognosis prediction. These results support the independence of this expression-based prognostic signature. Finally, we also examined the performances of our signature in CM considering less common mutations including NF-1 mutant, NRAS-mutant, and triple negative CM [26]. The results are shown in Fig. S1 and further support that our 7-RNA signature was independent of NRAS and NF-1 mutant status in BRAT-WT CM.

### Comparison of the signature with other known prognostic biomarkers

**Fig. 3:**
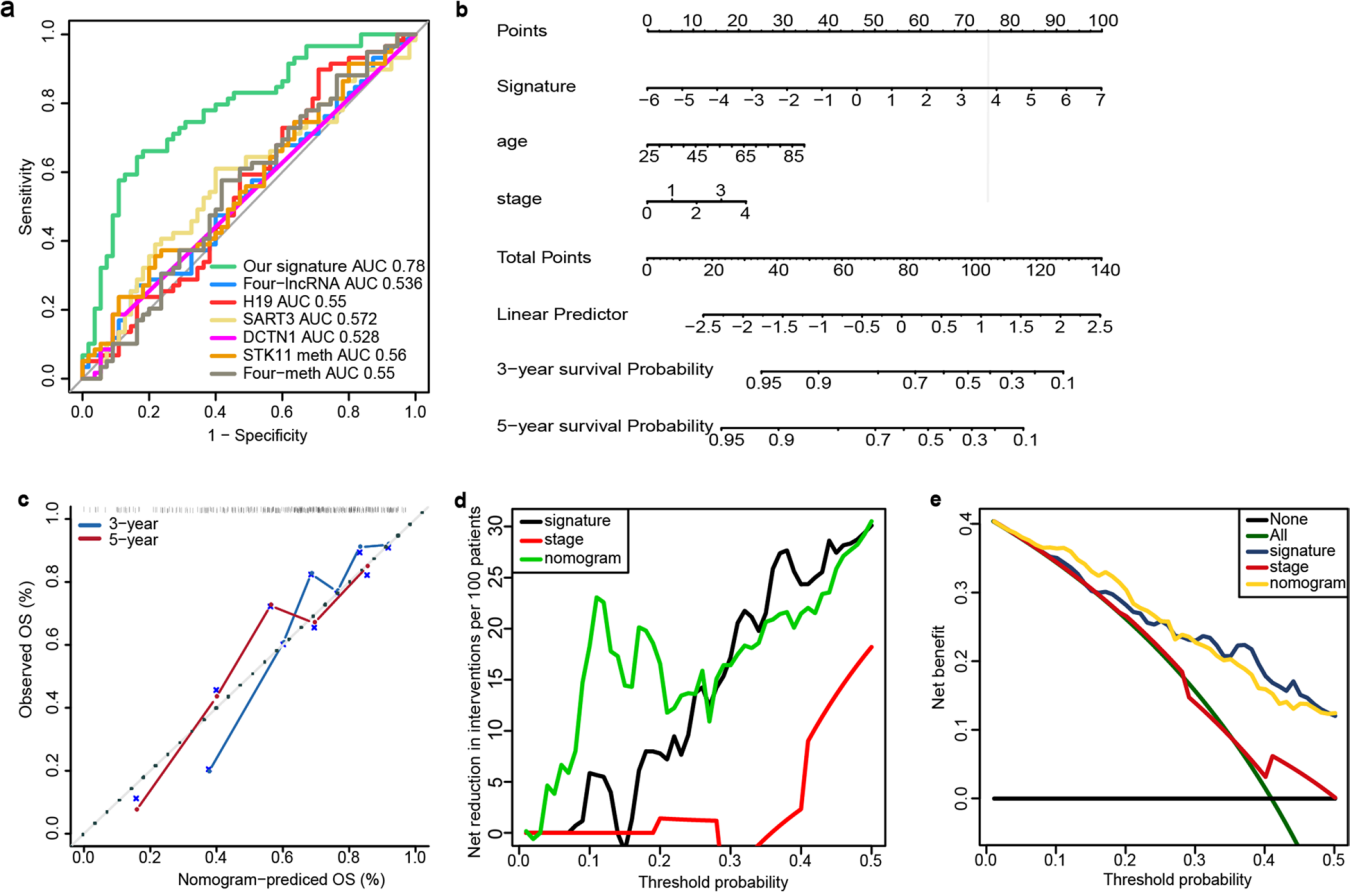
**a** Comparison of predictive performances of our 7-RNA signature and other known biomarkers/signatures. **b** The signature-based nomogram to predict 3-year and 5-year survival probability. **c** The calibration plot of nomogram predicted and real surviving proportions. **d** Net reduction interventions and (**e**) net benefit of decision curve analysis

Considering the burden brought by CM, several studies have proposed other biomarkers for CM prognosis prediction [8, 27–31]. Although none of them specifically aimed at WT Bf-CM, we compared our signature with them to examine the predictive performances in WT Bf-CM prognosis. As is shown in Fig. 3a and Table 3, the ROC analysis of time-dependent analysis in WT Bf-CM cohort proved that our signature had significant higher AUC than other biomarkers, and might be superior in WT Bf-CM prognosis prediction exclusively.

**Table 3:** The ROC results of our signature and other latest biomarkers of SKCM

Signature	AUC	95% CI of AUC	Type	*P*-value^a^	Reference
Our signature	0.780	0.70–0.87	LncRNA and mRNA		This study
Four-lncRNA	0.536	0.43–0.64	LncRNA	0.000	[26]
H19	0.550	0.44–0.66	LncRNA	0.000	[27]
SART3	0.572	0.47–0.68	Protein coding	0.002	[28]
DCTN1	0.528	0.46–0.60	Protein coding	0.000	[29]
STK11	0.560	0.45–0.67	Methylation	0.001	[30]
Four-DNA methylation	0.550	0.44–0.66	Methylation	0.002	[8]

^a^*P*-value of AUC value comparisons between our signature and other latest biomarkers

### Construction and evaluation of a prognostic nomogram for WT Bf-CM

Nomogram has been a welcomed quantitative predictive tool for clinical practice. Based on the results from univariable and multivariable analysis above (Fig. 2a), a nomogram to predict 3 − year and 5-year survival of WT Bf-CM patients was constructed based on the risk scores of the proposed signature, age and pathological stage (Fig. 3b). The C-index of this nomogram was calculated to be 0.7505 and calibration plot was also drawn, exhibiting an acceptable accuracy for prediction (Fig. 3c). Additionally, DCA was performed to assess the efficiency of the proposed nomogram (Fig. 3d, e). When the threshold probability was lower than 25%, our nomogram would be more beneficial and when threshold probability higher than 25%, our signature would be more beneficial. These results supported the promising complementary use of our signature and nomogram.

### Functional analysis based on the signature grouping

**Fig. 4:**
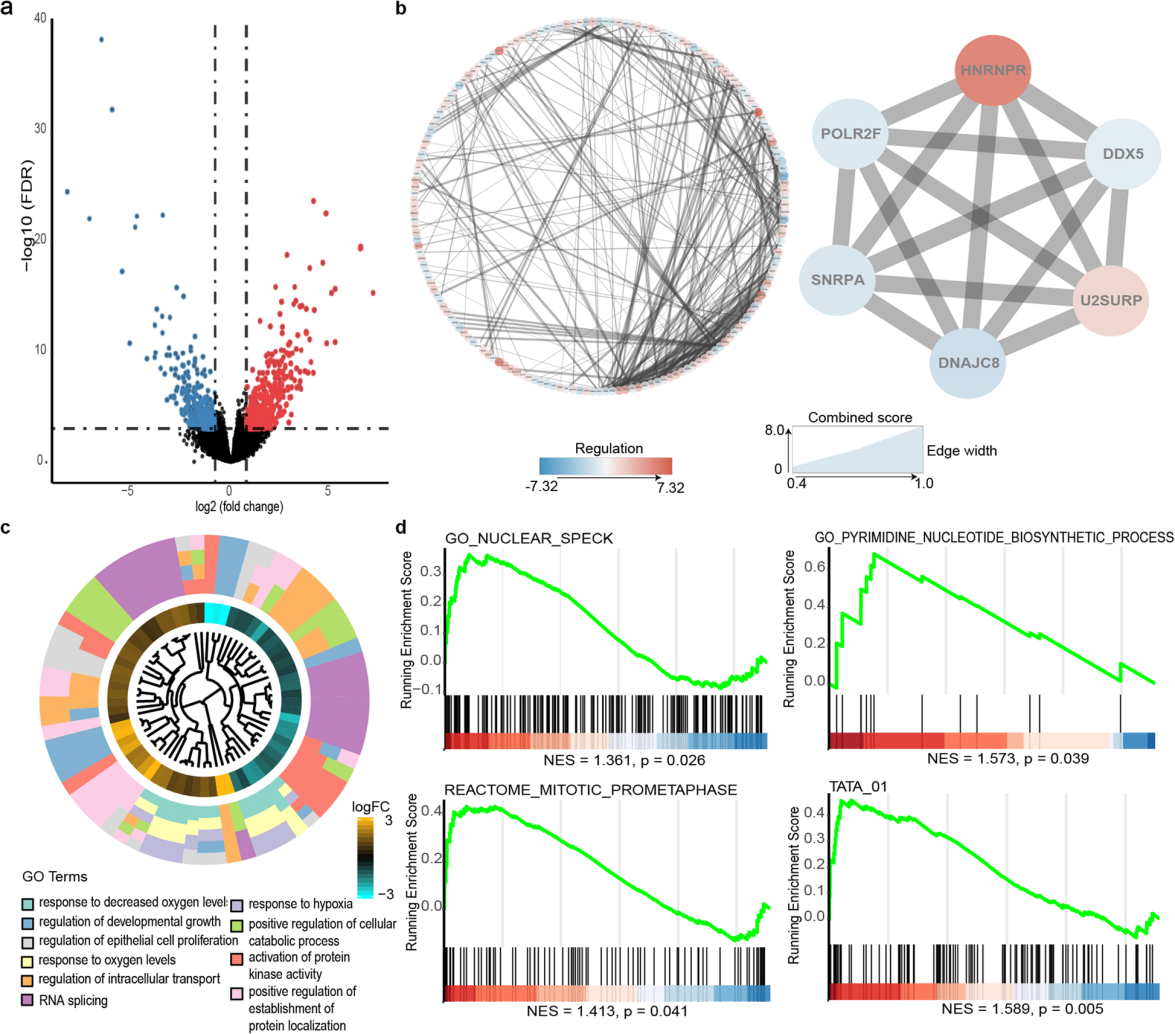
**a** Volcano plot of DEGs between high risk and low risk group. **b** Protein–protein interactions (PPI) of DEGs in String and core module of PPI with the highest Mcode score. **c** GOplot of BP enrichment results showing term-clustered gene set and fold changes of genes. **d** GSEA results of specific enrichment set in MSigDB

Cellular and molecular mechanism accounting for the prognosis of melanoma have always been a hot topic of cancer research. Thus, we aimed to find out what biological process might explain the predictive function of this signature. 865 genes were defined as DEGs between high risk and low risk group (Fig. 4a) and interactions of 527 proteins provided a landscape of potential molecular mechanisms (Fig. 4b). Using Mcode, a six-gene module with highest score (score 6) was selected as core module, which was closely associated with mRNA processing, splicing and metabolic process (Fig. 4b). BP enrichment analysis showed various processes were involved, including response to oxygen levels, RNA splicing, regulation of developmental growth, etc. (Fig. 4c). Besides, GSEA analysis indicated that the high-risk group was associated with the up-regulation of GO_NUCLEAR_SPECK (NES = 1.361, *p* = 0.026), GO_PYRIMIDINE_NUCLEOTIDE_BIOSYNTHETIC_PROCESS (NES = 1.573, *p* = 0.039), REACTOME_MITOTIC_PROMETAPHASE (NES = 1.413, *p* = 0.041) and TATA_01 (NES = 1.589, *p* = 0.005) (Fig. 4d). Taken together, these results supported that the predictive function of this signature might be related to the up-regulation of RNA splicing, transcription, and cellular proliferation in the high-risk group, and upregulation of these processes have been demonstrated to be linked to malignancy of cancer [32, 33].

### Evaluation for the therapeutic responses based on signature grouping

**Fig. 5:**
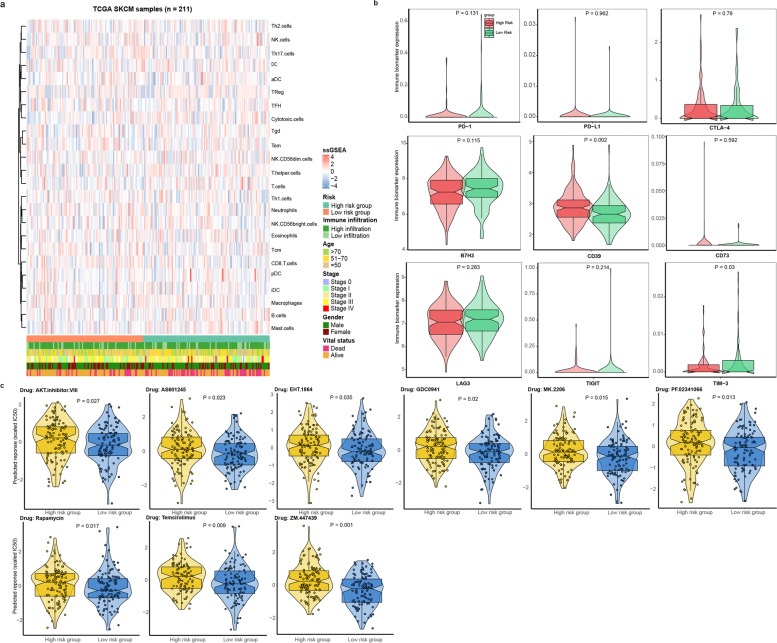
**a** Complex heatmap of 24 sorts of immune cells of WT Bf-CM patients, accompanied by signature-related risk groups, immune infiltration, age, stage, gender and vital status. **b** Expression of immune markers in high and low risk groups. **c** Predicted IC50 of various anti-cancer drugs. The *p* values were calculated using Wilcoxon rank-sum test

The treatment of cutaneous melanoma has made a considerable progress recently, especially for the application of immune-therapy and targeted drugs. Hence, we wondered if the proposed signature could provide clues for the therapeutic responses of WT Bf-CM patients. By estimating the immune infiltration of the WT Bf-CM cohort, we found that little differences in 24 sorts of immune cells infiltration (Fig. 5a). Similarly, the comparison of the expression of 9 immune-markers showed little differences between high- and low- risk groups (Fig. 5b). Of note, although the checkpoint therapies targeting programmed cell death 1 (PD-1), programmed cell death-ligand 1 (PD-L1) and cytotoxic T lymphocyte antigen 4 (CTLA-4) have been recommended for the treatment of WT Bf-CM, their expression didn’t seem to be high both in high- or low-risk groups, suggesting the poor responses of checkpoint therapy in WT Bf-CM patients. Interestingly, the analysis of responses to chemo-therapies showed significant differences of 9 chemotherapeutic drugs in estimated IC50 between high and low-risk groups, along with an increased IC50 to these 9 chemotherapies in high risk group (Fig. 5c). Among these 9 chemotherapeutic drugs, the targets of AKT.inhibitor.VIII, AS601245, GDC0941, MK.2206, PF.02341066 (Crizotinib), Rapamycin and Temsirolimus are involved in the regulation of PI3K/Akt/mTOR pathway [34–36], and this axis has known to play a critical role in cell proliferation and RNA processing [37, 38], further supporting the results from functional analysis. Altogether, these findings support that this signature could predict the responses of PI3K/Akt/mTOR pathway based drugs in WT Bf-CM patients, besides predicting their prognosis.

## Discussion

As a highly heterogenous cancer, CM harbours various genetic alteration including BRAF mutation, RAS mutation, NF1 mutations, etc., which plays an critical role in the biological behavior of CM and is closely related to the prognosis of CM patient [1]. However, existing studies often discussed the prognosis or proposed predictive signatures of the total CM patients, rather than focus on CM patients with specific genetic alteration [8, 27–31], which might fail to give a precise result. Since the mutation status of BRAF has been used as a prognosis biomarker, and the prognosis of WT Bf-CM seems relatively poor owing to the lack of therapeutic options [1–3, 39], this study aimed to construct a predictive signature for the prognosis of WT Bf-CM patients. After extracting and the whole transcription profiles of tumor tissues from 211 WT Bf-CM patients in the TCGA database, we constructed a 7-RNA based signature in the training set. According to the KM analysis and ROC analysis, this signature exhibited a considerable performance in prognosis prediction both in the test set (*p* = 0.005, AUC = 0.699) and entire set (*p* < 0.001, AUC = 0.780).

In order to ensure the independency for prediction, signature should be compared with clinical factors contributing to the prognosis with stratified analysis. However, little is known about clinical factors that contributes to the prognosis of WT Bf-CM patients. Using univariate and multivariate Cox analysis among classic clinical factors related to CM prognosis [25], we surprisedly found that only age and pathological stage were significantly correlated to the survival of WT Bf-CM patients. Although this might result from the limited size of our patients, it is of importance to make more exploration on the factors for WT Bf-CM prognosis. Subsequently, by stratifying the WT Bf-CM cohort according to age and pathological stage, the proposed signature was proven to be an independent predictive factor for the survival of WT Bf-CM prognosis. Next, by comparing the AUC curves, this signature exhibited a significant superior predictive performance exclusively for WT Bf-CM prognosis than other existing signatures for CM prognosis. In addition, to combine the predictive effect of this signature with clinical factors, a nomogram was drawn. Calibration and DCA analysis were performed and indicated a promising complementary use of our signature and nomogram. Given the poor understanding of factors associated with WT Bf-CM prognosis, the signature and nomogram in this study could make a more inspiring contribution to the precise management of WT Bf-CM.

Among the 7 RNA, CDC73 and TRIB2 have been identified as biomarkers for cancer prognosis [40, 41]. Especially, TRIB2 could be used to indicate progression and the response to chemotherapy in ex vivo clinical samples of melanoma [42]. As for the other 2 mRNA, VPS13D and CELF3, some multi-omics or pathways based analysis indicated the their roles as potential drug target or biomarker for metastasis, but more experimental evidences are needed to further confirm their molecular roles in cancer biology [43, 44]. Besides, little is known about the biological function of rest 3 ncRNA in cancer. Therefore, it is of significance to find some explanation of the biological mechanism for this signature. By comparing the transcription data between high- and low- risk groups, 865 DEGs were identified. Following analysis in protein interaction, biological process and pathway revealed that the up-regulation of mRNA splicing and processing, mitotic prometaphase, hypoxia-related metabolism alteration and pyrimidine nucleotide biosynthetic process were associated with the high-risk group. The former up-regulated processes are involved in the enhancement of cell proliferation and are linked to malignancy of cancer [33], which means the poor survival of WT Bf-CM predicted by this signature might be resulted from the more malignant behavior in cancer biology. This was supported by a retrospective study which concluded that low tumor proliferation rate was significantly associated with a better prognosis [45]. Moreover, in spite of the removal from sub-classification of thin melanomas in recent the AJCC staging system [46], mitotic rate can still be a significant prognostic indicator especially for WT Bf-CM.

As a cancer with high mortality, the survival of CM patients largely depends on the response to therapies. To date, immune checkpoint blockage therapy has been recommended for the treatment of advanced WT Bf-CM [47]. Additionally, the infiltration of immune cells can both serve as the predictor of responses to checkpoint therapy and WT Bf-CM survival [45, 48, 49]. However, little differences in immune infiltration was observed in the WT Bf-CM patients between high- and low- risk groups. Also, the expression of immune checkpoint such as PD-1, PD-L1 and CTLA-4, was relatively low expressed in WT Bf-CM patients, indicating the insensitivity of WT Bf-CM towards checkpoint therapy. Thus, we performed the analysis of responses to chemo-therapies and identified 9 drugs that might be effective for WT Bf-CM. Most of the 9 drugs function by targeting PI3K/Akt/mTOR axis. The axis of PI3K/Akt/mTOR, however, has been well-clarified as a key regulator in RNA processing, cellular proliferation and metabolic reprogramming, corresponding to the previous results in functional analysis in DEGs [37, 38, 50]. Three of the RNA for signature construction, including CDC73, TRIB2, VPS13D are, also involved in the regulatory network of PI3K/Akt/mTOR, which have been confirmed in functional experiments [43, 51, 52]. In the following evaluation for the therapeutic responses based on signature grouping, we could also infer a correlation between the PI3K/Akt/mTOR pathway and the prognosis we predicted via this signature, which corresponded with the functional analysis and further supported a critical role of PI3K/Akt/mTOR pathway in the prognosis of WT Bf-CM. Therefore, targeting the PI3K/Akt/mTOR pathway may be an important and promising target for the improvement of prognosis of WT Bf-CM.

Nevertheless, there are deficiencies with the present study. First, since it is difficult to find a cohort contain the corresponsive data of BRAF status, transcription and survival, this signature was not validated with external cohort. Second, simple cross validation might cause the problem of overfitting and weaken the ability of generalization. However, random forest is fundamentally based on independent model ensemble and adept in generalizing and avoiding overfitting. Insufficient trees were downvoted and testing set actually exerted a positive effect on selecting efficient results to eliminate out of bagging. Third, no experimental evidence supports the potential mechanisms of the present signature and further experiments might reveal the underlying molecular interactions.

## Conclusion

Collectively, this study proposed a novel 7-RNA signature for the prognostic prediction of WT Bf-CM. As far as we are concerned, this is the first prognostic signature for WT Bf-CM, which might provide more assistance in the management of WT Bf-CM patients.

## Supplementary Information

**Additional file 1: Figure S1.** KM analysis reveal the OS differences between high and low risk groups in NF-1 mutant (a), NRAS-mutant (b), and triple-wild-type (c) WT Bf-CM patients. ROC curves in corresponding subgroups (d-f) assess the predictive performance of the signature-related score. *P* values were calculated by two-sided log-rank tests, total AUC values were estimated and 95% CI were computed with 2000 stratified bootstrap replicates.

## Data Availability

All the data can be downloaded from https://portal.gdc.cancer.gov/.

## Abbreviations

WT Bf-CM: Wild-type BRAF cutaneous melanoma
CM: Cutaneous melanoma
PFS: Progression-free survival
NGS: Next-generation sequencing
RPKM: Reads Per Kilobase per Million mapped reads
OS: Overall survival
ROC: Receiver operating characteristic
AUC: The area under ROC
DCA: Decision curve analysis
DEG: Differently expressed genes
GESA: Gene set enrichment analysis
BP: Biological process
IC50: Inhibitory concentration 50
PD-1: Programmed cell death 1
PD-L1: Programmed cell death-ligand 1
CTLA-4: Cytotoxic T lymphocyte antigen 4
