# Supplementary material for: A novel 7 RNA-based signature for prediction of prognosis and therapeutic responses of wild-type BRAF cutaneous melanoma
Source: Biol Proced Online. 2022 Jun 24;24:7. doi: 10.1186/s12575-022-00170-2 (PMC9233353; doi:10.1186/s12575-022-00170-2)
Supplement: Supplementary file 1 — Additional file 1: Figure S1. KM analysis reveal the OS differences between high and low risk groups in NF-1 mutant (a), NRAS-mutant (b), and triple-wild-type (c) WT Bf-CM patients. ROC curves in corresponding subgroups (d-f) assess the predictive performance of the signature-related score. P values were calculated by two-sided log-rank tests, total AUC values were estimated and 95% CI were computed with 2000 stratified bootstrap replicates. [file 12575_2022_170_MOESM1_ESM.docx]

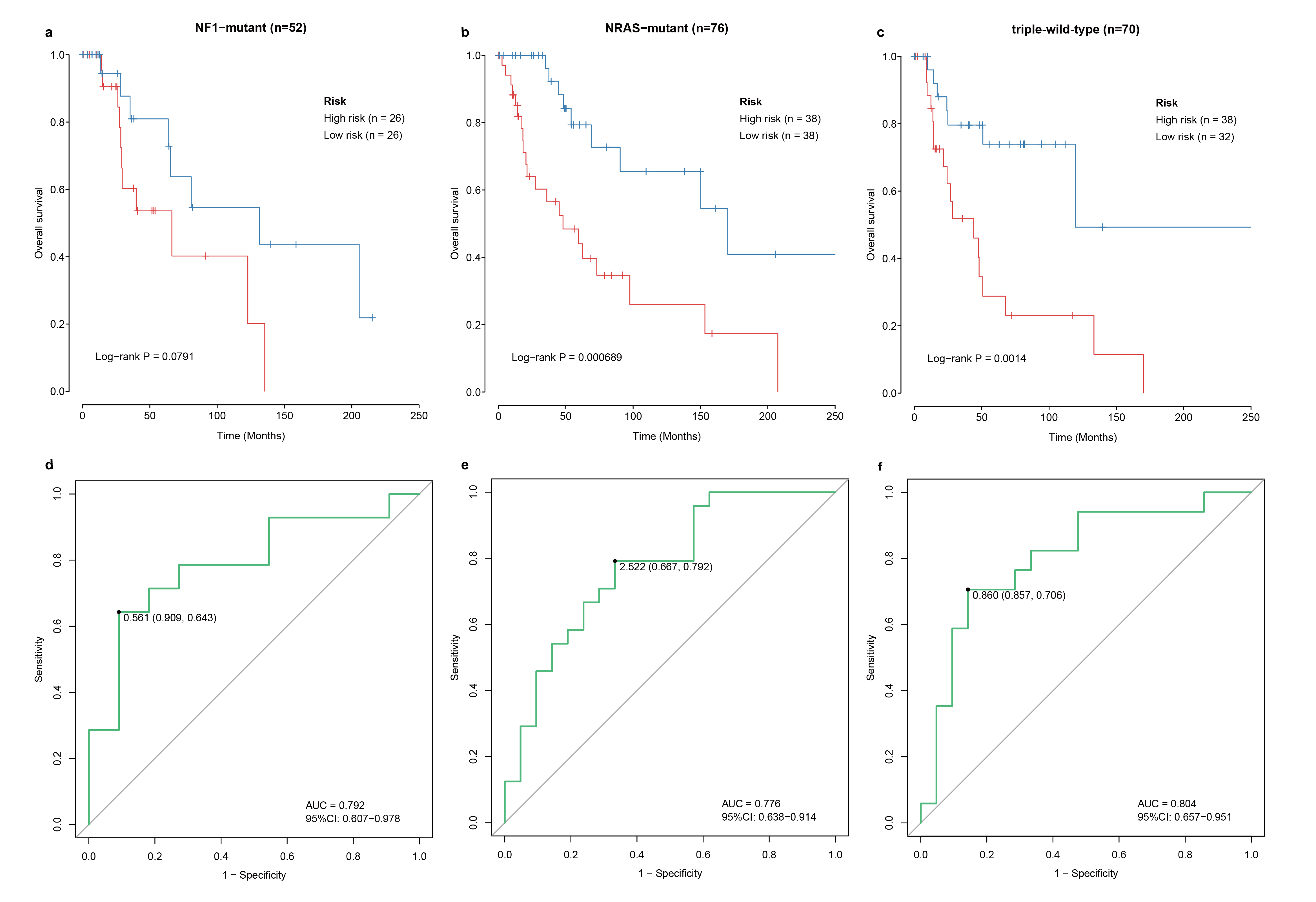


Figure S1: KM analysis reveal the OS differences between high and low risk groups in NF-1 mutant (a), NRAS-mutant (b), and triple-wild-type (c) WT Bf-CM patients. ROC curves in corresponding subgroups (d-f) assess the predictive performance of the signature-related score. P values were calculated by two-sided log-rank tests, total AUC values were estimated and 95% CI were computed with 2000 stratified bootstrap replicates.
